# Spatial Transcriptomic and miRNA Analyses Revealed Genes Involved in the Mesometrial-Biased Implantation in Pigs

**DOI:** 10.3390/genes10100808

**Published:** 2019-10-14

**Authors:** Ji Huang, Yifen Yang, Miao Tian, Dadong Deng, Mei Yu

**Affiliations:** Key Lab of Agricultural Animal Genetics, Breeding and Reproduction of Ministry of Education, College of Animal Science and Technology, Huazhong Agricultural University, Wuhan 430070, China; huang_ji@webmail.hzau.edu.cn (J.H.); yang-yifen@webmail.hzau.edu.cn (Y.Y.); tianmiao1211@webmail.hzau.edu.cn (M.T.); dadong_deng@163.com (D.D.)

**Keywords:** pig, endometrium, implantation, mesometrial, transcriptome, miRNA

## Abstract

Implantation failure is a major cause of early embryonic loss. Normally, the conceptus attachment is initiated at mesometrial side of the uterus and then spread to the anti-mesometrial side in pigs, however, the mechanisms that direct the mesometrial-biased attachment are largely unknown. In this study, the histological features of the entire uterine cross-section from gestational days 12 (pre-attachment stage) and 15 (post-attachment stage) were investigated and the differences in histological features between the mesometrial and anti-mesometrial side of the uterus were observed. Then, transcriptomic and miRNA analyses were performed on mesometrial and anti-mesometrial endometrium obtained from gestational days 12 and 15, respectively. Differentially expressed genes (DEGs) and miRNAs (DE-miRs) that were common to both or unique to either of the two anatomical locations of uterus were identified, respectively, indicating that differences in molecular response to the implanting conceptus exist between the two anatomical locations. In addition, we detected DEGs and DE-miRs between the two anatomical locations on the two gestational days, respectively. Of these DEGs, a number of genes, such as chemokine and T cell surface marker genes, were found to be significantly up-regulated mesometrially. Furthermore, we detected the interaction of *CXCR4*, *CXCL11* and miR-9 using dual luciferase reporter assay. Taken together, this study revealed genes and pathways that might play the role of creating a receptive microenvironment at the mesometrial side, which is required to guide a proper positioning of conceptus in the uterus in pigs.

## 1. Introduction

Failure in conceptus implantation is a major cause for the decrease in litter size in pigs. The porcine embryo freely migrates in the uterus between 4-cell stage to gestational day 13. Around gestational days 10–12, the conceptus undergoes dramatic changes in morphology. The initial attachment of porcine conceptus to the endometrium takes place around day 13 of gestation, forming a non-invasive epitheliochorial placenta [1,2,3]. In response to conceptus-derived factors, the endometrium undergoes remodeling and becomes receptive to the implanting conceptus. Previous studies have revealed the difference in expression patterns of gene, miRNA and lncRNA before and after the attachment stage in the endometrium in pigs [4,5,6,7,8]. A recent study conducted by using laser capture microdissection (LCM) determined the gene expression changes in different types of endometrial cells between day 12 of pregnant and non-pregnant cyclic gilts [9]. These findings identified genes and regulatory pathways that are associated with embryo implantation in pigs [10,11,12,13,14,15].

Blood vessels come into the uterus from the mesometrium to supply blood to the uterine horn, thus in the vertical direction, the uterus can be demarcated into mesometrial (M) and anti-mesometrial (AM) sides. Implanting of the floating embryo occurs towards particular side of the uterus. The embryo orientation is synchronized with the morphological changes within the uterine lumen. In mice, the blastocyst orients towards the anti-mesometrial side of the uterus and develops the placenta at the mesometrial side. The abnormalities in the establishment of this embryonic–uterine orientation will lead to aberrant implantation and embryo loss at the post-implantation stage [16]. Recently, uterine genes or pathways that have important contributions to this particular embryonic-uterine orientation have been characterized and the key role of these genes in regulating the embryo implantation has been determined [17]. In contrast to mice, normally, the initial attachment of the porcine conceptus in the uterus occurs at the mesometrial side [18]. In order to enable the initial contact, features in cell shape, secretion and adhesive properties are different in the uterine area to which the conceptus is close or attaches to as compared to the unapposed region [19,20,21]. However few studies have investigated the molecular mechanisms involved in the mesometrial-biased implantation in pigs. Tayade et al. reported that four genes (*VEGF*, *IFN-γ*, *TNF-α*, and *HIF-1α*), which are involved in angiogenesis or inflammatory responses, showed significantly different expression in endometrium between the mesometrial side and anti-mesometrial side of the uterus during peri-implantation in pigs [22]. Our previous study observed that expression of *ABCA1* protein was more intensive in the mesometrial endometrium of the porcine uterus [23]. These findings indicate that those uterine genes which exhibit different expression between the two anatomical locations (the mesometrial side and anti-mesometrial side of uterus) may be part of the conceptus orientating system. Therefore, investigation of those differentially expressed genes and their differential regulating mechanisms may enable us to explore the precise mechanisms of conceptus implantation in pigs.

In this study, the expression patterns of mRNA and miRNA in the endometrium at the mesometrial side and anti-mesometrial side of the uterus on gestational day 12 (before the initial physical attachment of conceptus to uterus) and day 15 (post-attachment of conceptus to uterus) were detected in pigs. Expression differences in mRNA and miRNA between the two anatomic locations were investigated. Then the miRNA-gene pairs that might be related to immune cell recruitment were identified.

## 2. Materials and Methods 

### 2.1. Samples Collection

All animal procedures were performed according to protocols approved by the Ethic Committee of Huazhong Agricultural University. Meishan gilts were obtained from the pig farm of Huazhong Agricultural University (Wuhan, China). Gilts were artificially inseminated twice after estrus. Uteri were obtained from gilts slaughtered on gestational days 12 (*n* = 3) and 15 (*n* = 6). Each uterus was cut into 10–15 cm pieces. Half of the uterine pieces were randomly selected and flushed with phosphate-buffered saline (PBS). When pregnancy was confirmed by the presence of filamentous conceptuses in uterine flushing, the endometrial tissues were taken from the mesometrial side and anti-mesometrial side of the uterus, respectively, frozen in liquid nitrogen and stored at −80 °C. Meanwhile, the rest of the uterine samples which were not flushed with PBS were fixed immediately in 10% neutral-buffered formalin for 24 h followed by paraffin embedding (FFPE).

### 2.2. Histological Analysis

The paraffin-embedded uterine samples were sectioned (4 μm thick), and stained with hematoxylin and eosin. Then the entire slide of uterine cross-section was scanned by a 3D HISTECH Pannoramic Midi scanner (3D HISTECH Ltd., Budapest, Hungary). The uterine cross-sectional images were taken and viewed by using CaseViewer software (3D HISTECH Ltd., Budapest, Hungary).

### 2.3. RNA-seq and miRNA-seq Data Analysis

Total RNA was isolated from the frozen endometrium tissue using the Trizol Reagent (Invitrogen Life Technologies), after which the concentration, quality and integrity were determined using a NanoDrop spectrophotometer (Thermo Scientific). Only samples with RNA integrity number > 7 were used for library construction. The library was then sequenced on a Hiseq platform (Illumina). Raw data and Clean data were obtained from Shanghai Personal Biotechnology Cp. Ltd. Reads were mapped to the porcine genome sequence assembly (*Sus scrofa* 11.1) with HISAT2 (v2.1.0) [24]. The mapped reads of each sample were assembled by HTseq [25]. The resulting read count tables were used to identify differentially expressed genes and miRNAs in DEseq2 [26]. Differentially expressed genes and miRNAs between the mesometrial side and anti-mesometrial side of the uterus were analyzed by using paired design, and differentially expressed genes and miRNAs between gestational days 12 and 15 were analyzed by using unpaired design. The adjusted *p*-values were determined according to false discovery rate (Benjamini-Hochberg) method. Gene ontology (GO) enrichment analyses of differentially expressed genes were explored by using DAVID Bioinformatics Resources 6.8 (https://david.ncifcrf.gov/) [27,28]. The target genes for the differentially expressed miRNAs were predicted by using TargetScan 7.2 (http://www.targetscan.org/vert_72/) [29].

### 2.4. Real-Time Polymerase Chain Reaction (RT-PCR) for Genes and miRNAs

Quantitative real-time polymerase chain reaction (qRT-PCR) was used to validate the RNA-seq and miRNA-seq data. The RNA samples were the same as we used for RNA-seq and miRNA-seq. The cDNA was obtained by using a PrimeScript RT Reagent Kit with a gDNA Eraser and One Step PrimeScript miRNA cDNA synthesis kit (Takara Bio Inc., Dalian, China) according to the manufacturer’s instructions. The qRT-PCR was performed using standard SYBR Premix Ex TaqII (Tli RNaseH Plus; Takara Bio) in a Bio-Rad CFX96 Touch Real-Time PCR Detection System (Bio-Rad Laboratories, Inc., Hercules, CA). PCR conditions were as follows: a single cycle of 30 s at 95 °C, followed by 39 cycles of 5 s at 95 °C and 30 s at 60 °C. The *GAPDH* gene was used as internal standard for genes, and U6 small nuclear RNA was used as internal standard for miRNAs. All of the primers used in the validation assays were listed in Supplementary Materials Table S1. A *p*-value of < 0.05 by *t*-test was considered to be significant.

### 2.5. Dual Luciferase Reporter Assays

Dual-luciferase reporter assay, which was used to validate the interaction relationship between miRNA and genes predicted by Targetscan, was carried out according to protocol reported previously [30]. Wild-type or mutant 3′UTR fragments of CXCR4 and CXCL11 were synthesized by Tsingke Biotech (Wuhan, China) and inserted into the multiple cloning sites of psi-CHECK-2 (Promega, Madison, WI, USA). The miR-9 mimic, their mutant miRNA mimics (miR-9_mut) and scrambled sequence (NC) were synthesized as duplexes (sequences are listed in Supplementary Materials Table S1). For the luciferase reporter assay, PK15 cells were co-transfected with the reporter vectors, miRNA mimics, mutant miRNA mimics, or NC by using Lipofectamine 2000 (Invitrogen). After 24 h, cells seeded in 96-well plates were collected and measured by using the Dual-Luciferase Reporter Assay System (Promega, Madison, WI, USA). The transfections were repeated three times, and three replicates were performed for each transfection. A *p*-value of <0.05 by *t*-test was considered to be significant. 

## 3. Results

### 3.1. Analysis of the Histological Features of the Whole Cross-Sectional View of the Uterus during Implantation in Pigs

**Figure 1:**
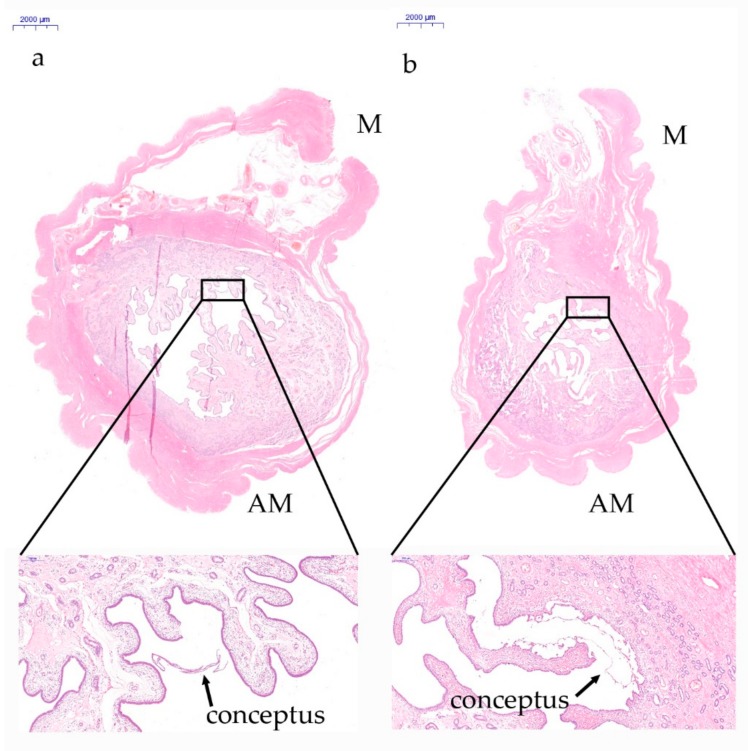
Photomicrographs of representative uterine sections obtained from gestational days 12 and 15 in pigs and stained with hematoxylin and eosin. (**a**) On gestational day 12, the filamentous conceptus migrates toward the mesometrial side of the uterus. (**b**) On gestational day 15, the conceptus attaches at the mesometrial side of uterus. M, mesometrial side of uterus. AM, anti-mesometrial side of uterus.

In this study, we first investigated the characteristics of whole uterine cross-sectional images taken from gestational days 12 and 15, respectively. As shown in Figure 1, the floating conceptus migrates toward the mesometrial side of the uterus on gestational day 12 and is in contact with the uterine endometrium at the mesometrial side on gestational day 15. In addition, compared to the mesometrial endometrium, the anti-mesometrial endometrium extends greatly to the uterine cavity, resulting in different histological features between the two anatomical positions in the porcine uterus during implantation.

### 3.2. Gene Expression Patterns before and after Conceptus Attachment Differed between the Mesometrial and Anti-Mesometrial Endometrium in the Uterus

The transcriptome data were generated for endometrial tissues from the mesometrial side and anti-mesometrial side of porcine uterus on gestational days 12 and 15, respectively. About 580 million clean reads per sample (Supplementary Materials Table S2) were generated and all the cleaned reads were assembled into a total of 25,580 genes. 

**Figure 2:**
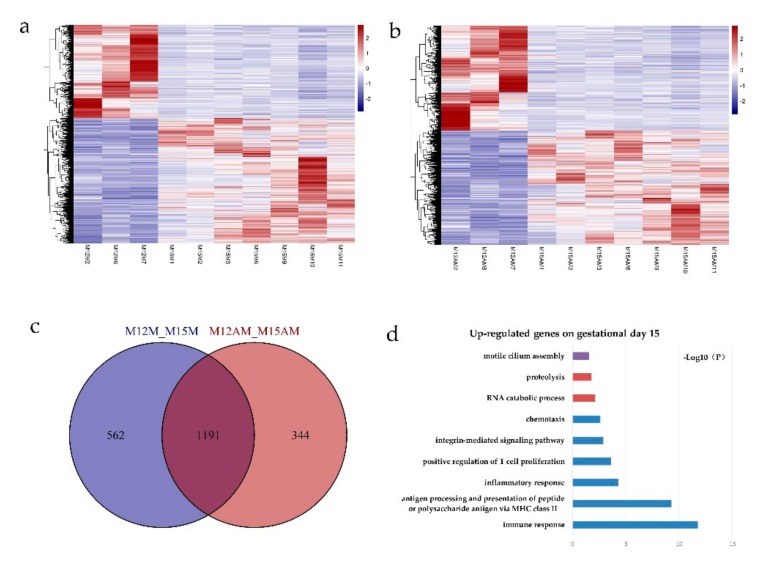
Gene expression profiles between the mesometrial side and anti-mesometrial side of uterus during implantation. (**a**) Expression profiles of the differentially expressed genes at the mesometrial side of the uterus between gestational days 12 and 15; (**b**) Expression profiles of the differentially expressed genes at the anti-mesometrial side of uterus between gestational days 12 and 15. Each row represents an individual gene, and each column represents a sample. The color legend at the top indicates the level of gene expression. (**c**) Venn diagram showing the overlaps of differentially expressed genes (DEGs) between the mesometrial side and anti-mesometrial side of uterus between gestational days 12 and 15. (**d**) Gene Ontology (GO) analysis of the up-regulated DEGs on gestational day 15. Blue column represents GO terms for DEGs that were common to both the mesometrial and anti-mesometrial side, red column represents GO terms for the DEGs that were unique to the mesometrial side, and purple column represent GO terms for DEGs that were unique to the anti-mesometrial side. The negative log of the P-value (-log10P) was plotted on the x-axis. M12M, gestational day 12 at the mesometrial side of the uterus; M12AM, gestational day 12 at the anti-mesometrial side of the uterus; M15M, gestational day 15 at the mesometrial side of the uterus; M15AM, gestational day 15 at the anti-mesometrial side of the uterus.

**Figure 3:**
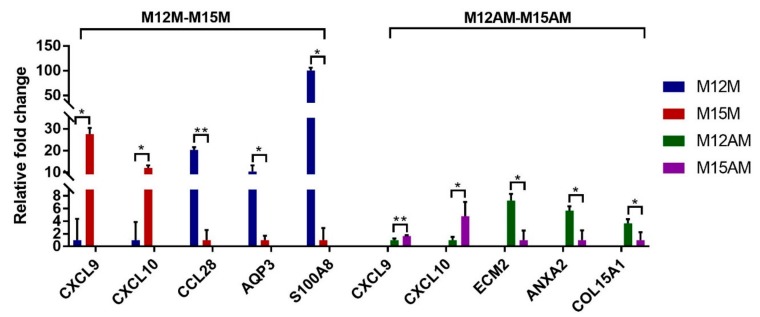
Validation of the differentially expressed genes between gestational days 12 and 15 in the mesometrial endometrium and anti-mesometrial endometrium, respectively. The error bars showed the standard deviation (SD). The *p*-value was calculated using the *t*-test. * *p* < 0.05; ** *p* < 0.01. M12M, gestational day 12 at the mesometrial side of the uterus; M12AM, gestational day 12 at the anti-mesometrial side of the uterus; M15M, gestational day 15 at the mesometrial side of the uterus; M15AM, gestational day 15 at the anti-mesometrial side of uterus.

Transcriptome analysis revealed that a total of 1753 genes were differentially expressed (DEGs) between gestational days 12 and 15 in the mesometrial endometrium (1002 up-regulated and 751 down-regulated genes; adjusted *p*-value < 0.05, |fold change| > 2, basemean > 100; Figure 2 and Supplementary Materials Table S3), while a total of 1535 genes were differentially expressed between gestational days 12 and 15 in the anti-mesometrial endometrium (801 up-regulated and 734 down-regulated genes; adjusted *p*-value < 0.05, |fold change| > 2, basemean > 100; Figure 2 and Supplementary Materials Table S3). The expression patterns of these DEGs were analyzed by qRT-PCR. As shown in Figure 3, the qRT-PCR results were in agreement with the RNA-seq data. Of the DEGs between gestational days 12 and 15, a total of 1191 DEGs were commonly expressed in endometrial tissues from both the mesometrial and anti-mesometrial side, and a total of 562 DEGs and 344 DEGs were unique to endometrial tissues from the mesometrial side and anti-mesometrial side, respectively (Figure 2). Gene ontology (GO) analysis of the up-regulated DEGs on gestational day 15 revealed that the most significant GO terms for the common DEGs include immune response, antigen processing and presentation of peptide or polysaccharide antigen via MHC class II, inflammatory response, and integrin-mediated signaling pathway. The most enriched GO terms for the DEGs that were unique to the mesometrial side were involved in proteolysis and the RNA catabolic process, while those GO terms for the DEGs that were unique to the anti-mesometrial side were involved in motile cilium assembly (Figure 2). The results indicate that during the early gestational stages, regulation of immune related genes was required for endometrial remodeling at both the mesometrial and anti-mesometrial side of the uterus, and gene expression patterns before and after conceptus attachment were different between the mesometrial and anti-mesometrial endometrium in the uterus.

### 3.3. Endometrial Genes Exhibit Spatial Expression Patterns in the Uterus

We then compared the transcriptome profiles for the endometrial tissues between the two anatomical location (the mesometrial side and anti-mesometrial side of the uterus). On day 12 of gestation, only 5 genes were differentially expressed (3 up-regulated and 2 down-regulated genes; adjusted *p*-value < 0.05, |fold change| > 1.5, basemean > 100; Supplementary Materials Table S4). While on day 15 of gestation, a total of 240 DEGs were detected (183 up-regulated and 57 down-regulated genes; adjusted *p*-value < 0.05, |fold change| > 1.5, basemean > 100; Supplementary Materials Table S4). 

**Figure 4:**
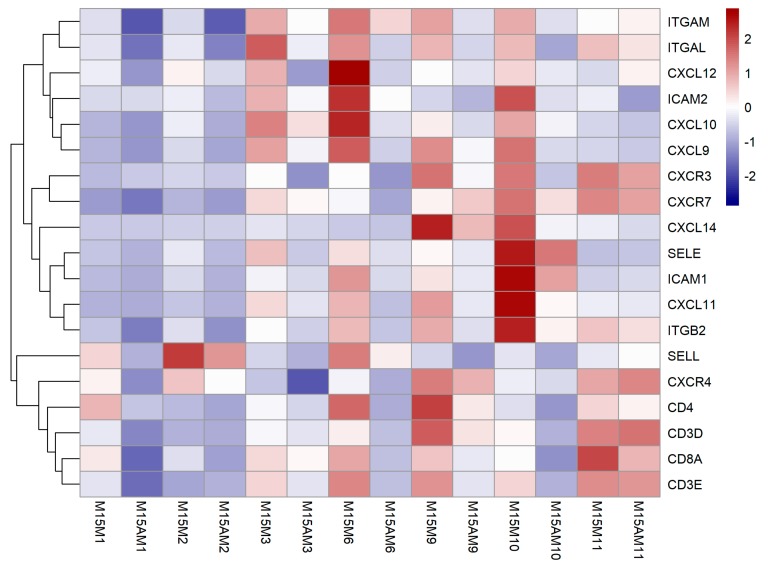
Heatmap for the immune cell recruitment-associated candidate genes (adjusted *p*-value < 0.05) which were up-regulated at the mesometrial side of uterus on gestational day 15. Each row represents an individual gene, and each column represents a sample. The color legend at the top indicates the level of gene expression, in which red represents a high expression level and blue a low expression level. M15M, gestational day 15 at the mesometrial side of the uterus; M15AM, gestational day 15 at the anti-mesometrial side of the uterus.

**Figure 5:**
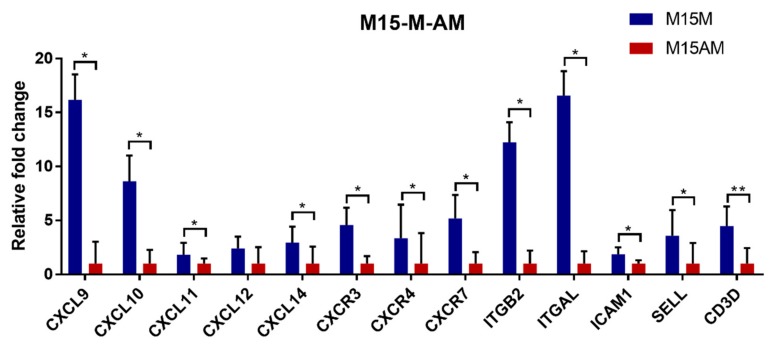
Validation of the differentially expressed genes between the mesometrial and anti-mesometrial side of the uterus at gestational day 15 by qRT-PCR. The error bars show the SD. The *p*-value was calculated using the *t*-test. * *p* < 0.05; ** *p* < 0.01. M15M, gestational day 15 at the mesometrial side of the uterus; M15AM, gestational day 15 at the anti-mesometrial side of the uterus.

Further analysis revealed that most of these DEGs detected on gestational day 15 are immune-related genes. It has been suggested that chemokines promote the recruitment of various immune cells into the endometrium by binding to their receptors during the implantation period in pigs [31,32]. Our RNA-seq data and qRT-PCR results showed that chemokine genes (*CXCL9*, *CXCL10*, *CXCL11*, *CXCL12*, *CXCL14* and their receptor *CXCR3*, *CXCR4*, *CXCR7*) were up-regulated in the endometrium at the mesometrial side (Figure 4 and Figure 5). LFA-1 was found on circulating leukocytes which was composed of *ITGAL* and *ITGB2*. *ICAM-1* was found on endothelial cells, which was a ligand for *LFA-1*. Circulating leukocytes could bind to endothelial cells via tight junctions of *LFA-1* and *ICAM-1* [33]. We found that the *ITGB2*, *ITGAL* and *ICAM1* were also up-regulated in the endometrium at the mesometrial side on day 15 of gestation (Figure 4). Consistent with the expression patterns of these chemokine genes, several T cell surface marker genes including genes coding for the two chains (*CD3D* and *CD3E*) of the CD3 molecule were observed to be up-regulated in the endometrium at the mesometrial side on day 15 of gestation (Figure 4).

### 3.4. Identification of Spatiotemporal Endometrial miRNA Expression Profiles in the Uterus during Implantation

**Figure 6:**
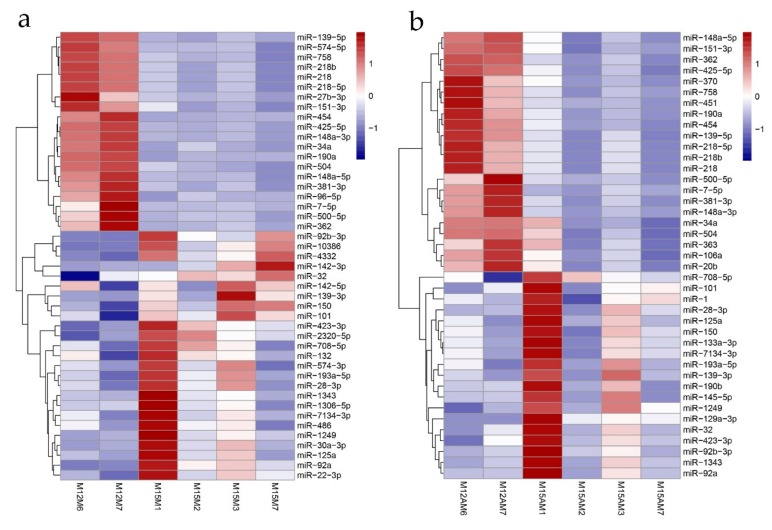
MiRNA expression profiling between the mesometrial side and anti-mesometrial sides of uterus at gestational days 12 and 15. (**a**) Expression profile of the differentially expressed miRNAs at the mesometrial side of the uterus between gestational days 12 and 15; (**b**) Expression profile of the differentially expressed miRNAs at the anti-mesometrial side of the uterus between gestational days 12 and 15. Each row represents an individual miRNA, and each column represents a sample. The color legend at the top indicates the level of miRNA expression. M12M, gestational day 12 at the mesometrial side of the uterus; M12AM, gestational day 12 at the anti-mesometrial side of the uterus; M15M, gestational day 15 at the mesometrial side of the uterus; M15AM, gestational day 15 at the anti-mesometrial side of the uterus.

**Figure 7:**
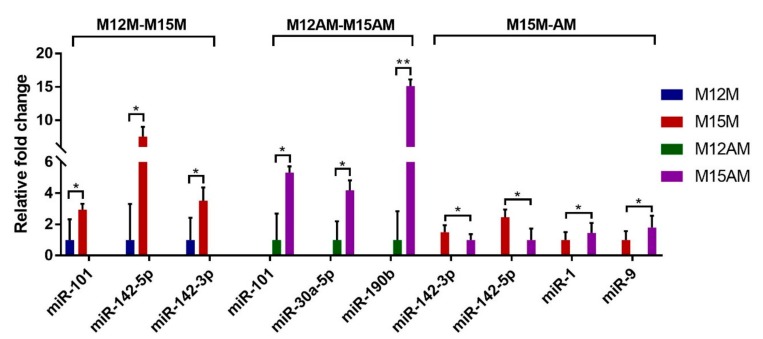
Validation of the differentially expressed miRNAs in porcine endometrium between gestational days 12 and 15. The error bars show the SD. The *p*-value was calculated using the *t*-test. * *p* < 0.05; ** *p* < 0.01. M12M, gestational day 12 at the mesometrial side of the uterus; M12AM, gestational day 12 at the anti-mesometrial side of the uterus; M15M, gestational day 15 at the mesometrial side of the uterus; M15AM, gestational day 15 at the anti-mesometrial side of the uterus.

A total of 45 miRNAs were detected to be differentially expressed between gestational days 12 and 15 in the mesometrial endometrium (25 up-regulated and 20 down-regulated miRNAs; adjusted *p*-value < 0.05, |fold change| > 2, basemean > 100; Figure 6 and Supplementary Materials Table S5), and 41 miRNAs were differentially expressed between gestational days 12 and 15 in the anti-mesometrial endometrium (19 up-regulated and 22 down-regulated miRNAs; adjusted *p*-value < 0.05, |fold change| > 2, basemean > 100; Figure 6 and Supplementary Materials Table S5). Of these differentially expressed miRNAs, a total of 31 miRNAs were commonly expressed in the mesometrial and anti-mesometrial endometrium. In addition, a total of 14 and 10 miRNAs were differentially expressed in either mesometrial or anti-mesometrial endometrium. In addition, we detected one miRNA (miR-142-5p) that was differentially expressed in endometrium between the two anatomical locations of uterus on gestational day 15 (adjusted *p*-value < 0.05, basemean > 100; Supplementary Materials Table S6). The relative expression levels of 7 differentially expressed miRNAs were analyzed by qRT-PCR and as shown in Figure 7. As compared to day 12 of gestation, miR-101, miR-142-5p and miR-142-3p were up-regulated in the mesometrial endometrium from day 12 to 15 of gestation, and miR-101, miR-30a-5p and miR-190b were up-regulated in the anti-mesometrial endometrium from day 12 to 15 of gestation. In addition, miR-142-5p was confirmed to be up-regulated in the mesometrial endometrium compared to the anti-mesometrial side on day 15 of gestation. The results were in agreement with our miRNA-seq data. Meanwhile, qRT-PCR was also conducted on 3 miRNAs (miR-142-3p, miR-1 and miR-9) whose expressions were not identified to be significant difference between the two anatomical locations by miRNA-seq. The results showed they were differentially expressed between the two anatomic locations on day 15 of gestation, in which miR-142-3p was up-regulated in the mesometrial endometrium, whereas miR-1 and miR-9 were down-regulated in the mesometrial endometrium.

### 3.5. Validation of the Predicted Interaction of miRNA-Target Pairs

**Figure 8:**
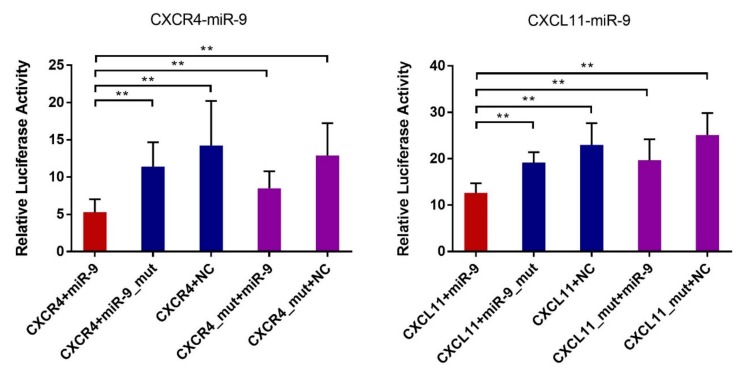
Validation of the predicted miRNA-target interactions with the 3′UTR luciferase reporter system. The wild-type 3′UTR reporter plasmids or mutant 3′UTR plasmids were co-transfected into the PK15 cells in combination with the miRNA mimics, scrambled sequence (NC), or mutant miRNA mimics, respectively. The y-axis shows the dual luciferase activity ratio (Renilla/Firefly luciferase). The error bars show the SD. ** *p* < 0.01.

In order to identify miRNAs that might regulate the differential expression of the immune cell recruitment-associated candidate genes between the two anatomical location (the mesometrial side and anti-mesometrial side of the uterus) on gestational day 15, we predicted the putative binding sites within the 3′UTR of these genes for the miRNAs that were up-regulated at anti-mesometrial side by using TargetScan. The results showed that the 3′UTR of both CXCR4 and CXCL11 contains putative binding sites of miR-1 and miR-9. Dual luciferase reporter assays were performed to validate the predicted miRNA-mRNA interactions. The predicted interaction between miR-1 and the 3′UTRs of CXCR4 and CXCL11 were not validated in this study. However, as shown in Figure 8, luciferase activity was significantly reduced by transfection of a wild-type miR-9 mimic with a plasmid containing corresponding binding sequence compared to transfection of NC, whereas transfection with a mutant miR-9 mimic or a plasmid containing mutant seed region failed to decrease luciferase activity. The results confirmed the potential binding between miR-9 and the 3′UTR of CXCR4 and CXCL11. 

## 4. Discussion

After being resealed from the zona pellucida, the pig blastocyst migrates freely in the uterus from one horn to the other and undergoes elongation. Normally, the initial conceptus attachment happens at mesometrial side of the uterus in pigs [2,18]. The differences in morphology and gene expression between the mesometrial side and anti-mesometrial sides of the uterus were reported in several studies, implying a role of uterine genes in guiding proper conceptus orientation for successful implantation [19,23]. In this study, we investigated for the first time the genome-wide gene and miRNA expression profiles in endometrial tissues from the two anatomical positions in the uterus (mesometrial side and anti-mesometrial side of uterus) around the conceptus attachment phase in pigs.

In this study, differentially expressed genes between gestational days 12 (before attachment) and 15 (post-attachment) in the mesometrial and anti-mesometrial endometrium were identified, respectively. Most of the DEGs were common to both sides and these common DEGs were enriched in GO terms of immune response, which is in agreement with previous reports [6,34]. The results suggest that during the early gestational stages, regulation of immune-related genes is required for endometrial remodeling at both the mesometrial and anti-mesometrial sides of the uterus. In addition, DEGs that were unique to mesometrial or anti-mesometrial endometrial tissues were also detected, implying that differences in response to the implanting conceptus might exist between the mesometrial and anti-mesometrial endometrium in the uterus. 

By comparing gene expression profiles between the mesometrial and anti-mesometrial endometrium at the two gestational days, respectively, we identified a few number of DEGs on day 12 of gestation, but the number of DEGs increased on day 15 of gestation. Pig conceptuses begin to release estrogen into the uterine lumen between gestational days 11 to 12 as the major signal for maternal recognition of pregnancy [21]. The attachment of the conceptus to the uterus is initiated at the mesometrial side on day 13 of gestation in pigs. In response to the conceptus signal, normally, the mesometrial endometrium starts to become receptive in order to guide the mesometrial-biased conceptus adhesion [18]. Our findings revealed that, coinciding with this process, endometrial genes exhibited different expression patterns between the mesometrial side and anti-mesometrial side of the uterus, and the differences became apparent as implantation progresses. Thus, these DEGs might contribute to guiding a proper positioning of porcine conceptus in the uterus.

Around the period of attachment phase, a delicate immunological balance between the maternal and embryonic immune system has to be established at the implantation site [35]. The implantation of the conceptus influences the distribution of immune cells in uterine [36,37]. Altered maternal immunological response could lead to rejection of the implanting conceptus. Nevertheless how these immune cells are recruited to the implantation site is not entirely understood in porcine endometrium [35]. Leukocyte recruitment to sites of inflammatory site has been described as a multi-step processes governed by chemokines, selectins and integrin that engage in a step-wise manner to initiate intracellular signals and adhesive bond formation [38]. Our data revealed that several chemokine genes (*CXCL9, CXCL10, CXCL11, CXCL12, CXCL14* and their receptor *CXCR3, CXCR4, CXCR7*), selectin genes (*SELL* and *SELE*), integrin genes (*ITGB2, ITGAL, ITGAM, ICAM1* and *ICAM2*) were significantly up-regulated at the mesometrial side of uterus relative to the anti-mesometrial side on gestational day 15. These findings suggest that a chemotactic gradient might exist over the uterine wall with a higher concentration in the mesometrial endometrium, to which the conceptus attaches. On the other hand, we found that several T cell surface marker genes showed the same expression pattern as those leukocyte recruitment-related genes. In addition, TCR/CD3 complex is a multi-chain structure contributing to antigen recognition and signal transduction in T-cell activation [39]. The genes coding for two chains of TCR/CD3 complex were detected to exhibit higher expression levels in endometrium from mesometrial side compared to anti-mesometrial side. Thus the results imply that those leukocyte-recruitment related genes might play a key role in the process of recruiting immune cells to the maternal-fetal interface. 

MicroRNAs (miRNAs) are small endogenous non-coding RNAs that regulate gene expression by inducing mRNA degradation or inhibiting mRNA translation [40]. Previously, Krawczynski and Li reported a number of miRNAs that were expressed in endometrial tissues during implantation in different pig breeds [4,8]. In this study, the miRNA profiles were investigated in the same endometrial samples used for RNA-seq. In agreement with the previous reports, we found that miRNAs (such as miR-1, miR-9, miR-30a-5p, miR-92a, miR-101, miR-142-3p, miR-142-5p, miR-148a-3p) were highly expressed in porcine endometrium tissues. In addition, our study detected miRNAs that were expressed differentially in endometrial tissues before and after the conceptus attachment, in which those common to both or unique to either of the two anatomical locations of uterus were identified, respectively. Notably, several miRNAs were identified to be differentially expressed in the endometrium between the two anatomic locations of uterus on gestational day 15. The findings suggest that the alteration of miRNA levels might contribute to, at least in part, the differences in gene expression pattern we detected between the mesometrial side and anti-mesometrial side of uterus.

Among the miRNAs differentially expressed between gestational days 12 and 15, miR-142-5p and miR-142-3p exhibited a negative expression pattern with *ITGB*8, which could influence endometrial receptivity and regulate embryo attachment [41,42]. The 3′UTR region of *ITGB*8 has a binding site for miR-142-3p, and this regulatory relationship has been reported in glioma [43]. In this study, the regulatory relationship between porcine *ITGB*8 and miR-142-3p was validated by dual luciferase reporter assay, indicating that miR-142-3p might affect the embryonic attachment process via regulating *ITGB*8 in pigs (Figure S1). Of the miRNAs that were expressed differentially in endometrial tissues between the mesometrial and anti-mesometrial sides of the uterus, miR-9 showed a negative expression pattern with *CXCR*4 and *CXCL*11. Several articles have reported the targeting regulation between miR-9 and *CXCR*4 in different tumors, nevertheless the targeting relationship between miR-9 and *CXCL*11 has not been reported [44,45]. Our results of dual luciferase reporter assay confirmed that miR-9 can target porcine *CXCR*4 and *CXCL*11, suggesting a role of miR-9 in recruitment immune cells during implantation by regulating genes of *CXCR*4 and *CXCL*11 in pigs.

## 5. Conclusions

In summary, this study comprehensively revealed spatiotemporal endometrial gene and miRNA expression profiles in the porcine uterus during implantation. Notably, the differentially expressed genes and miRNAs were identified between the mesometrial side and anti-mesometrial side of the uterus. In addition, we found a lot of immune cell recruitment-associated genes were up-regulated at the mesometrial side to which the conceptus attaches, and miRNA that might regulate expression of these genes were characterize. Taken together, our results provided candidate genes and pathways that may have a key role in regulating the mesometrial-biased implantation in pigs.

## Supplementary Materials

The following are available online at https://www.mdpi.com/2073-4425/10/10/808/s1: Table S1: Primer list of differentially expressed genes and miRNAs validation, miRNA mimics sequences and scrambled sequence (NC) in dual luciferase reporter assays. Table S2: Raw data statistic of RNA-seq. Table S3: List of differentially expressed genes between gestational days 12 and 15. Table S4: List of differentially expressed genes between the two anatomical location (the mesometrial side and anti-mesometrial side of uterus). Table S5: List of differentially expressed miRNAs between gestational days 12 and 15. Table S6: List of differentially expressed miRNAs between the two anatomic location (the mesometrial side and anti-mesometrial side of the uterus). Figure S1: Validation of the predicted miR-142-3p-ITGB8 interactions with the dual luciferase reporter system.
