# Supplementary material for: Spatial Transcriptomic and miRNA Analyses Revealed Genes Involved in the Mesometrial-Biased Implantation in Pigs
Source: Genes (Basel). 2019 Oct 14;10(10):808. doi: 10.3390/genes10100808 (PMC6826901; doi:10.3390/genes10100808)
Supplement: Supplementary file 1 [file genes-10-00808-s001.zip › Final Supplementary File/Figure S1.docx]

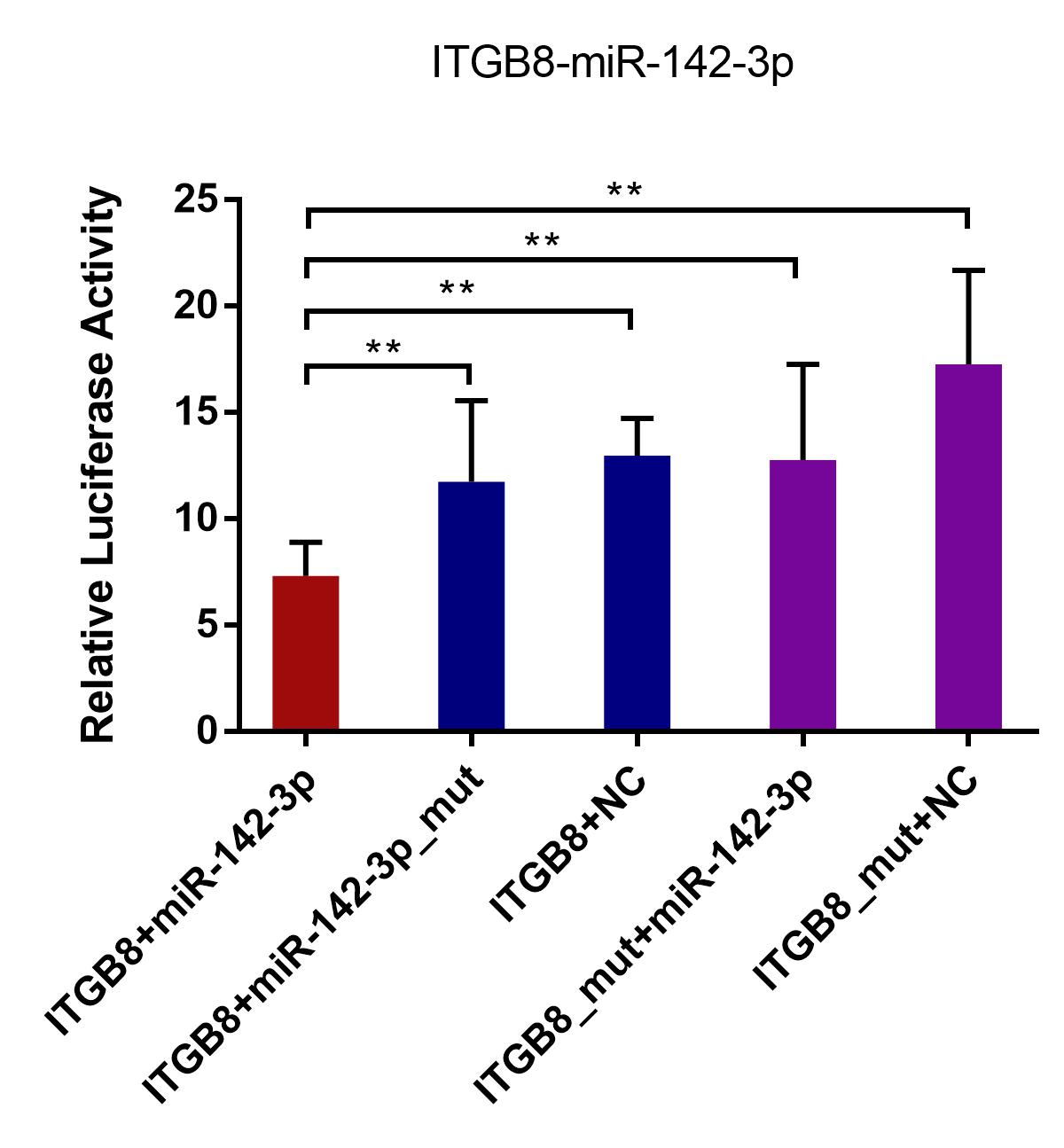


**Figure S1.** Validation of the predicted miRNA-target interactions with the 3’UTR luciferase reporter system. The wild-type 3’UTR reporter plasmids or mutant 3’UTR plasmids were co-transfected into the PK15 cells in combination with the miRNA mimics, NC, or mutant miRNA mimics, respectively. The y-axis shows the dual luciferase activity ratio (Renilla/Firefly luciferase). The error bars show the SD. ***P* <0.01.
