# Supplementary figures and images for: Spatial Transcriptomic and miRNA Analyses Revealed Genes Involved in the Mesometrial-Biased Implantation in Pigs
Source: Genes (Basel). 2019 Oct 14;10(10):808. doi: 10.3390/genes10100808 (PMC6826901; doi:10.3390/genes10100808)

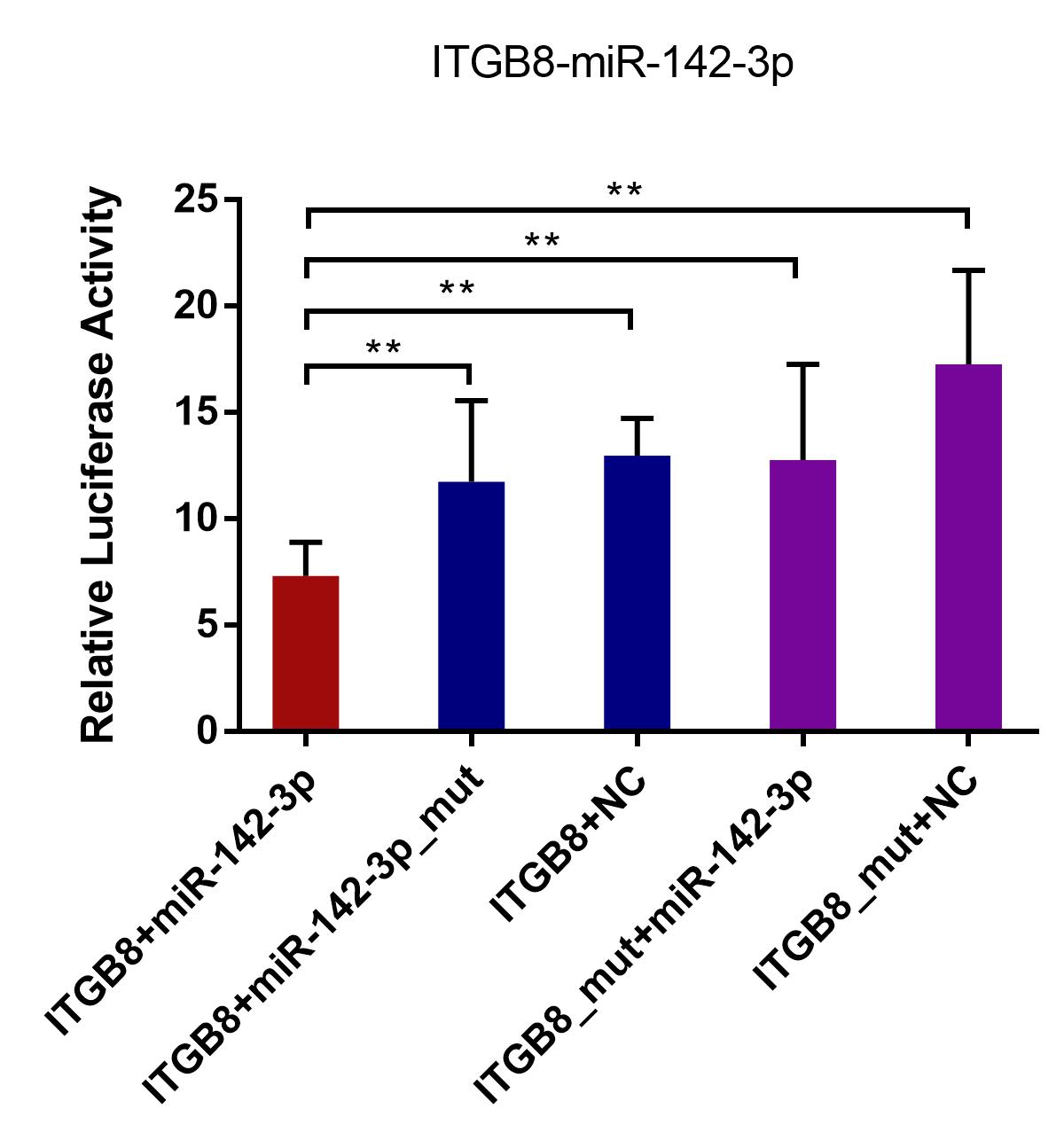

Supplement: Supplementary file 1 [file genes-10-00808-s001.zip › Final Supplementary File/Figure S1.jpg]
